# The G protein‐coupled estrogen receptor of the trigeminal ganglion regulates acute and chronic itch in mice

**DOI:** 10.1111/cns.14367

**Published:** 2023-07-14

**Authors:** Jun Li, Po Gao, Siyu Zhang, Xiaoqi Lin, Junhui Chen, Song Zhang, Yingfu Jiao, Weifeng Yu, Xiaoqiong Xia, Liqun Yang

**Affiliations:** ^1^ Department of Anesthesiology Chaohu Hospital Affiliated to Anhui Medical University Hefei Anhui China; ^2^ Department of Anesthesiology Renji Hospital, Shanghai Jiao Tong University School of Medicine Shanghai China; ^3^ Key Laboratory of Anesthesiology (Shanghai Jiao Tong University) Ministry of Education Shanghai China; ^4^ Department of Anesthesiology The Second Affiliated Hospital of Jiaxing University Zhejiang China

**Keywords:** G protein‐coupled estrogen receptor, itch, trigeminal ganglion, TRPA1, TRPV1

## Abstract

**Aims:**

Itch is an unpleasant sensation that severely impacts the patient's quality of life. Recent studies revealed that the G protein‐coupled estrogen receptor (GPER) may play a crucial role in the regulation of pain and itch perception. However, the contribution of the GPER in primary sensory neurons to the regulation of itch perception remains elusive. This study aimed to investigate whether and how the GPER participates in the regulation of itch perception in the trigeminal ganglion (TG).

**Methods and Results:**

Immunofluorescence staining results showed that GPER‐positive (GPER^+^) neurons of the TG were activated in both acute and chronic itch. Behavioral data indicated that the chemogenetic activation of GPER^+^ neurons of the TG of *Gper‐Cre* mice abrogated scratching behaviors evoked by acute and chronic itch. Conversely, the chemogenetic inhibition of GPER^+^ neurons resulted in increased itch responses. Furthermore, the GPER expression and function were both upregulated in the TG of the dry skin‐induced chronic itch mouse model. Pharmacological inhibition of GPER (or *Gper* deficiency) markedly increased acute and chronic itch‐related scratching behaviors in mouse. Calcium imaging assays further revealed that *Gper* deficiency in TG neurons led to a marked increase in the calcium responses evoked by agonists of the transient receptor potential ankyrin A1 (TRPA1) and transient receptor potential vanilloid V1 (TRPV1).

**Conclusion:**

Our findings demonstrated that the GPER of TG neurons is involved in the regulation of acute and chronic itch perception, by modulating the function of TRPA1 and TRPV1. This study provides new insights into peripheral itch sensory signal processing mechanisms and offers new targets for future clinical antipruritic therapy.

## Graphical abstract

The GPER of TG plays a crucial role in modulating acute and chronic itch signaling. Pharmacological activation of GPER and chemogenetic activation of GPER‐positive neurons result in increased itch responses. Conversely, *Gper* deficiency markedly increases acute and chronic itch‐related scratching behaviors in mouse, and the underlying molecular mechanisms may be related to the functional upregulation of TRPV1 and TRPA1.

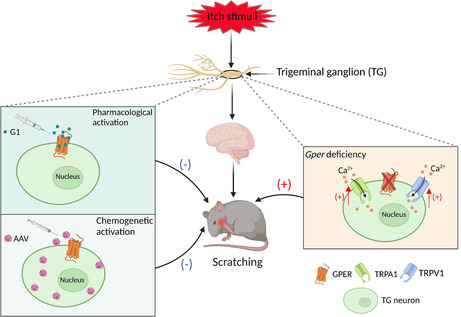

## 1 INTRODUCTION

Itch, was first defined in the 1600s as “an unpleasant sensation that evokes a desire or reflex to scratch.”^1^ While pain evokes an immediate avoidance of danger, itch triggers a scratching response, indicating that the organism is suffering from an external injurious irritation (such as the itch caused by a mosquito bite) or certain diseases.^2^ In pathological states, this acute sensation may turn into chronic pruritus, which is often accompanied by skin diseases,^3^ endocrine and metabolic diseases,^4^ and mental disorders.^5^ Long‐term chronic itch affects around 15% of the global population, with a significant negative impact on sleep quality, mental health, and the patient's overall quality of life.^1^ However, in contrast to pain, no drugs to treat itch are currently approved by the Food and Drug Administration (FDA) of the United States.^6^, ^7^ Therefore, exploring the mechanisms of pruritus and searching for its potential therapeutic targets is of general interest to researchers and clinicians.

In patients with pruritic dermatitis, sudden or recurrent facial itch is one of the most frequently observed symptoms.^8^ Previous studies have demonstrated the essential roles of estrogen and estrogen receptors in facial itch sensation.^9^, ^10^ In female patients, some pruritic skin disorders show variations in itch sensation during different stages of the menstrual cycle.^11^ During the pediatric age, the prevalence of atopic dermatitis appears relatively higher in boys than in girls; however, this gender difference is reversed after puberty.^12^, ^13^ These clinical observations may be due to periodic changes in estrogen levels. The dynamic effects of these changes could affect the occurrence of itch in inflammatory skin diseases.^14^ The specific molecular mechanisms associated with these diseases are unclear and may involve the modulation of itch perception by estrogen, through its binding to high‐affinity estrogen receptors.^15^, ^16^

Itch is evoked by skin receptors that perceive external stimuli and generate impulses. Sensory neurons in the dorsal root ganglion (DRG) and trigeminal ganglion (TG) transmit itch signals to the spinal cord or trigeminal spinal tract nuclei, and eventually to the cerebral cortex.^17^, ^18^ The TG is an important coding site for itch sensation on facial skin.^19^, ^20^ Many ion channels and receptors play important roles in the production and transmission of itch perception. These include the transient receptor potential vanilloid V1 (TRPV1) and the transient receptor potential ankyrin A1 (TRPA1), which mediate histaminergic and non‐histaminergic itch signals, respectively.^21^, ^22^ It has been demonstrated that the G protein coupled estrogen receptor (GPER) is expressed in TG neurons.^23^ G protein‐coupled estrogen receptor is a seven‐transmembrane G protein‐coupled receptor (GPCR) that specifically binds estrogen and propagates intracellular signals.^24^, ^25^ Our recent study demonstrated that the GPER upregulation in the rostral ventromedial medulla (RVM) inhibited itch sensation, and the associated downstream pathway could be related to μ opioid receptor phosphorylation.^26^ Formononetin can alleviate the atopic dermatitis‐generated itch perception through the GPER‐mediated upregulation of A20 protein levels.^27^ Therefore, the GPER may contribute to the regulation of itch perception. The TG could function as a direct link to convey the facial sensory signals to the central brain regions.^19^, ^20^ As such, we speculate that the GPER in TG neurons may be involved in the regulation of facial itch biochemical signals.

In this study, we aimed to identify the role and uncover the molecular mechanisms of acute and chronic itch perception by the GPER of the TG. First, we detected the activation of GPER‐positive neurons and the GPER expression in the TG, under acute and chronic itch conditions. Then, using chemogenetic methods, we investigated the functional role of GPER‐positive neurons in *Gper‐Cre* mice. Furthermore, using *Gper* knockout mice, we assessed whether the GPER in TG neurons mediates itch sensation. Finally, we examined the effects of the *Gper* deletion on the functions of TRPA1 and TRPV1, through calcium imaging. In summary, we found that the GPER plays a crucial role in modulating acute and chronic itch perception in the TG. The underlying molecular mechanisms may involve the functional modulation of TRPA1 and TRPV1.

## 2 MATERIALS AND METHODS

### 2.1 Animals

Adult male C57BL/6J mice (Shanghai Jiesjie Laboratory Animal Co., Ltd), *Gper‐Cre* transgenic mice (Shanghai Biomodel Organism Science Co., Ltd), and *Gper* knockout (*Gper* ^−/−^) mice (Bioray Biotechnology Co., Ltd) were utilized in the experiments. The mice were housed (5 animals per cage) in a temperature‐controlled (22–25°C) and humidity‐controlled (50%) room with a 12‐h light/12‐h dark cycle and had ad libitum access to food and water. The animal experiments were approved by the Ethics Committee for Experimental Use of Animals of the Shanghai Jiao Tong University School of Medicine (approval code: #SYXK‐2013‐0050) and were conducted in compliance with the Guiding Principles in the Care and Use of Animals and the Animal Management Rule of the Ministry of Public Health, China (documentation 545, 2001). Considering the potential effects of the estrus cycle on itch‐related scratching behaviors, female mice were not used for behavioral assays in this study.

### 2.2 Acute chemical itch

Behavioral tests of acute itch were performed as in previous studies.^28^, ^29^ Three days before the behavioral test, mice were shaved on the cheek. They were placed into small transparent plastic chambers (18 × 14 × 12 cm^3^) for routine training (three consecutive days; 30 min per day). On the day of the behavioral test, mice were acclimated in the test chamber for 30 min. Histamine (100 μg/10 μL) and chloroquine (50 μg/10 μL) were injected intradermally into the cheek. Immediately after the injection, single mouse was placed inside the observation boxes and videotaped for 30 min. The number of hindpaw scratch bouts at the injection sites was counted by an investigator who was blinded to the genotypes and treatments.

### 2.3 Chronic itch model

To induce chronic itch on the mouse cheek, a dry skin itch model was constructed using acetone, ether, and water (AEW).^30^ Briefly, the mouse cheeks were shaved before the treatment. The shaved area was treated daily with cotton soaked in a 1:1 mixture of acetone and ether for 15 s, followed by cotton soaked in distilled water for 30 s. Mice were treated twice a day (at 9 a.m. and 5 p.m.) for 5 days. The negative control was treated with cotton soaked in distilled water for 45 s. Then, on the morning of Day 6, single mouse was placed into the behavioral test environment to acclimatize for 30 min, and then spontaneous scratch bouts were recorded for 60 min with a digital camera. The investigator who counted the number of spontaneous scratch bouts was blinded to the genotypes and treatments of the mice.

### 2.4 Retrograde labeling

Cholera toxin subunit B (CTB), a widely used nontoxic retrograde tracer,^31^, ^32^ labels nerve cells with high specificity and efficiency, through its binding to the ganglioside pentosan chain (GM1) attached to the cell surface.^33^ To trace the projection of TG neurons into the facial skin, mice were briefly anesthetized with isoflurane and CTB‐488 (1 μg/μL, BrainVTA) was injected intradermally into three sites of the cheek. To prevent leakage, the injection site was gently pressed for 30 s. Tissue collections were performed 7 days after the injection.

### 2.5 Immunofluorescence staining

Mice were sacrificed with an overdose of sodium pentobarbital and further perfused with a saline solution, followed by a 4% paraformaldehyde (PFA) solution. The TGs were carefully removed and postfixed overnight at 4°C in 4% PFA. For cryoprotection, they were sequentially incubated in 20% and 30% sucrose solutions. Using a cryotome (Leica, Germany), samples were cut into 10 μm thickness sections and further mounted on glass slides. Sections were incubated with 10% normal goat serum and 1% Triton X‐100 for 1 h, followed by overnight incubation at 4°C with primary antibodies. Primary antibodies used in these experiments were rabbit anti‐GPER (1:1000, Lifespan), rabbit anti‐c‐Fos (1:500, Abcam), mouse anti‐neuroflament‐200 (NF200; 1:1000, Abcam), mouse anti‐calcitonin gene‐related peptide (CGRP; 1:100, Abcam), mouse anti‐isolectin B4 conjugated to fluorescein isothiocyanate (IB4‐FITC; 1:1000, Sigma), rabbit anti‐TRPA1 (1:500, Abcam), and guinea pig anti‐TRPV1 (1:1000, Millipore). Then, sections were incubated for 2 h at room temperature with secondary antibodies (Alexa Fluor 405, goat anti‐guinea pig; Alexa Fluor 488, goat anti‐rabbit or goat anti‐mouse; Alexa Fluor 594, donkey anti‐rabbit, 1:1000, Abcam). For antibodies of the same species, the Tyramide Signal Amplification (TSA) Biotin System kit (Thermo Fisher Scientific) was used for staining, as previously described.^34^, ^35^ To exclude nonspecific staining, a negative control was used for each immunofluorescence staining experiment, consisting in omitting the primary antibody. Finally, fluorescent images were observed with a laser‐scanning confocal microscope (Olympus Corporation). Analysis and counting were performed with the ImageJ software. For each TG, at least 10 randomly selected sections were recorded. For each animal, at least 500 TG neurons were counted. Cell counts were manually performed in a blinded manner.

### 2.6 Hematoxylin and eosin staining

After the mice were sacrificed, the cheek skin was immediately removed and postfixed by overnight immersion in a 4% PFA solution. Sections were dehydrated in ethanol, embedded in paraffin, and stained with hematoxylin and eosin (HE). Sections were visualized and photographed using a microscope (Leica). The epidermal thickness of the mouse skin was measured.

### 2.7 Quantitative RT–PCR analysis

For quantitative PCR (qPCR) on a LightCycler 480 II (Roche) system, total TG RNA was extracted with an RNA Extraction Kit (EZBioscience), cDNA was synthesized with an RNA Reverse Transcription Kit (Vazyme) and further mixed with SYBR Green PCR mix (EZ Bioscience). The primer sequences (5′–3′) used for cDNA amplification were as follows: Gper, CCTGCTACTCCCTCATCG (forward) and ACTATGTGGCCTGTCAAGGG (reverse); Gapdh, AAGAAGGTGGGTGACAGGCATC (forward) and CGGCACATCGGAGGAATG (reverse). We chose Gapdh as the loading control and applied the 2−ΔΔCT method to calculate the relative expression levels of the Gper mRNA.

### 2.8 Western Blot

Freshly isolated TGs were used for total protein extraction. After quantification, total proteins were separated by electrophoresis and transferred to a polyvinylidene fluoride (PVDF) membrane. Then, after being blocked with 5% nonfat milk for 1 h, samples were incubated with primary antibodies for 24 h at 4°C, including anti‐GPER (1:1000, ABclonal) and anti‐GAPDH (1:20,000, Proteintech). After incubation with the secondary antibody (horseradish peroxidase‐conjugated anti‐IgG antibody), protein bands were visualized with a chemiluminescence reagent (Thermo). The density of the bands was quantified using the ImageJ software.

### 2.9 TG stereotaxic injection

The trigeminal injection was performed following previous studies.^36^, ^37^ *Gper‐Cre* mice were anesthetized with pentobarbital (50 mg/kg, i.p.) and secured in a stereotaxic frame. After a midline incision on the scalp, the skull was exposed and drilled. The virus, such as rAAV‐hSyn‐DIO‐hM3D(Gq)‐mCherry‐WPRE‐hGHpA or rAAV‐hSyn‐DIO‐hM4D(Gi)‐mCherry‐WPRE‐hGHpA (BrainVTA), was injected into the TG using a microinjection needle. To allow adequate virus spread, the needle was kept inserted for 10 min after injection. We confirmed the successful virus injection by fluorescence microscopy observation of the TGs.

### 2.10 Cell dissociation and culture

TG neuron culture was performed as described in previous studies.^38^, ^39^ For washing and mincing, freshly isolated TGs were collected in precooled Hanks' balanced salt solution (HBSS, Gibco). Cells were digested with Liberase TM (0.35 U/mL, Roche) for 20 min at 37°C, followed by an HBSS enzyme solution containing Liberase TL (0.25 U/mL, Roche), papain (30 U/mL, Worthington Biochemical), and 0.6 mM EDTA for 20 min at 37°C. Then, cells were gently triturated and separated in a medium containing bovine serum albumin (1 mg/mL, Sigma) and a trypsin inhibitor (1 mg/L, Sigma). After centrifugation, TG cells were resuspended in freshly prepared medium. TG neurons were then plated on glass coverslips (Fisherbrand) precoated with poly‐D‐lysine (0.1 mg/mL, Sigma) and laminin (5 μg/mL, Sigma). Cells were cultured at 37°C and were used within 24 h.

### 2.11 Intracellular calcium imaging

TG neurons on glass slides were incubated with the fluorescent indicator fura‐2‐AM (5 μM, Invitrogen) for 30 min at 37°C, in the dark. Then, slides were transferred into a recording chamber. Images were acquired using an inverted fluorescence microscope system (DMI8, Leica) with alternating excitation wavelengths of 340 and 380 nm. The 340 nm/380 nm fluorescence intensity ratio [*R*(340/380)], measured with the PCI software every 2 s, was applied as an intracellular calcium concentration ([Ca^2+^]i) indicator.^40^ If the increase in [*R*(340/380)] was equal to or greater than 15% of baseline, neurons were considered to be responsive to a particular chemical stimulus. At the end of each assay, a 50 mM K^+^ solution was used to test neuronal viability. The concentrations of the following chemical compounds were determined according to previous studies^41^, ^42^, ^43^: G1 (GPER agonist, 10 μM, Cayman Chemical), chloroquine (CQ, 1 mM, Sigma), histamine (His, 100 μM, Sigma), allyl isothiocyanate (AITC, 100 μM, Sigma) and capsaicin (Cap, 1 μM, Sigma).

### 2.12 Statistical analysis

All data are presented as the mean ± standard error of the mean (SEM). Statistical analysis was performed using GraphPad Prism 9.0 (GraphPad Software). Data normality was verified by Shapiro–Wilk test. To compare differences between the two groups, the unpaired Student's *t*‐test was used. For multiple group comparisons, one‐way ANOVA followed by Tukey's post hoc test was used. To compare proportions, the chi‐squared (χ^2^) test was used. Differences were considered statistically significant when a *p‐*value was lower than 0.05.

## 3 RESULTS

### 3.1 The GPER is expressed in mouse face‐innervating TG neurons

**FIGURE 1:**
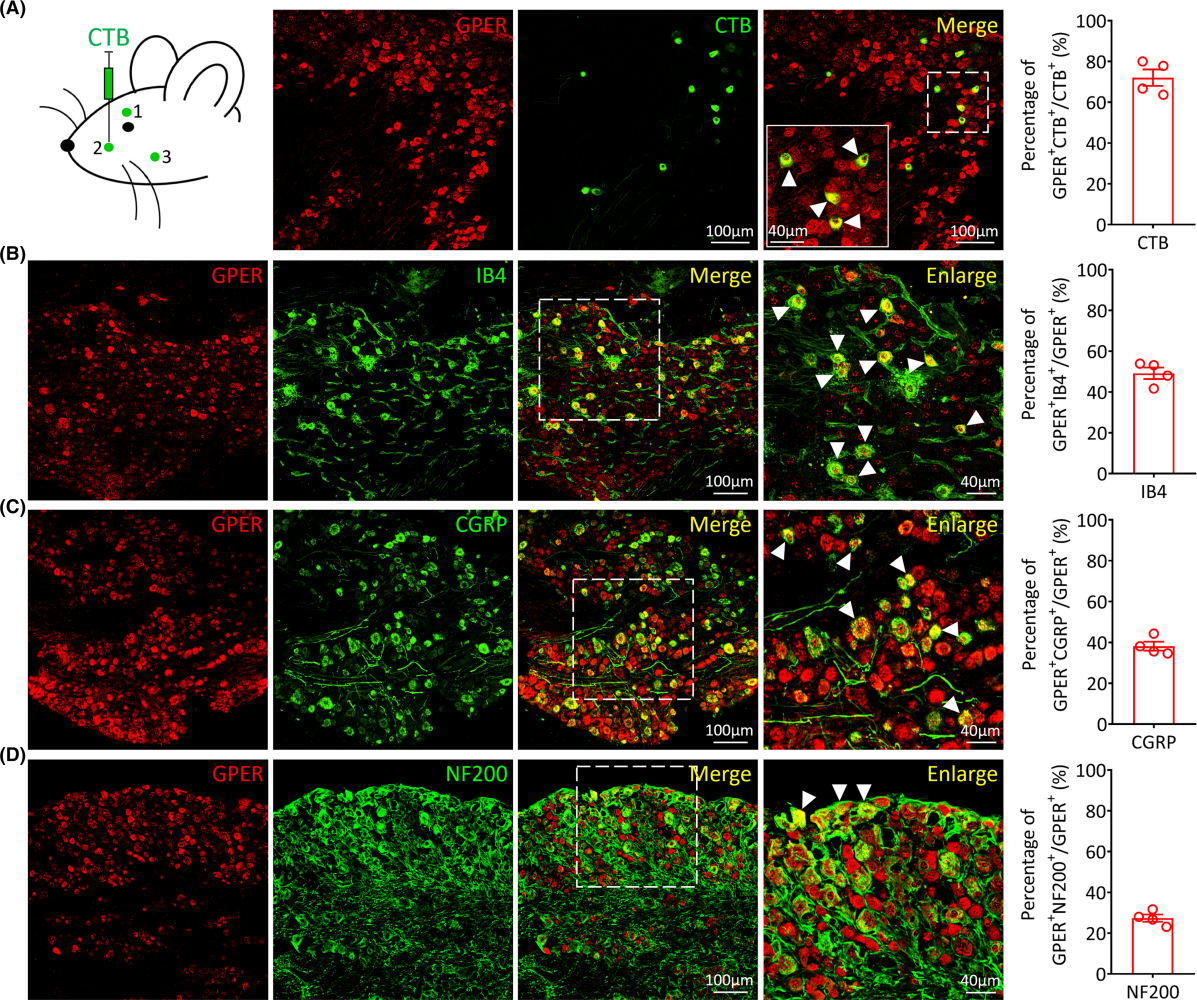
Expression and distribution of the G protein‐coupled estrogen receptor (GPER) in trigeminal ganglion (TG) neurons of the mouse. (A) Schematic diagram of cholera toxin subunit B (CTB) injection in mouse and representative immunofluorescence images of CTB‐labeled TG neurons innervating the mouse cheek colocalized with GPER, along with the quantitative analysis of the overlap percentage. (B–D) Double immunofluorescence staining showing the colocalization of GPER with IB4 (B), CGRP (C) and NF200 (D), along with quantitative analysis of the overlap percentage (*n* = 4 mice per group, scale bar: 100 or 40 μm).

First, to investigate whether GPER‐positive (GPER^+^) neurons of the TG can innervate the face, we injected the retrograde tracer CTB into the face of mice. The results showed that 72.02 ± 4.04% of the CTB‐positive TG neurons were GPER‐positive (Figure 1A), suggesting that GPER^+^ neurons of the TG can innervate the cheek region. A previous study has shown that the GPER is primarily expressed in the small‐diameter neurons in the rat TG.^44^ To characterize the cellular distribution of the GPER in TG neuron subpopulations, we used double immunofluorescence staining to assess the colocalization of the GPER with IB4 (a nonpeptidergic nociceptive marker), CGRP (a peptidergic nociceptive marker), and NF200 (a marker for large‐diameter neurons). The results showed that 49.17 ± 2.82% of the GPER^+^ neurons colocalized with IB4 (Figure 1B), 38.15 ± 2.17% of the GPER^+^ neurons colocalized with CGRP (Figure 1C), and 27.36 ± 1.77% of the GPER^+^ neurons colocalized with NF200 (Figure 1D). These results imply that the GPER in the TG is mainly localized in small‐diameter nociceptive neurons.

### 3.2 GPER ^+^ neurons in the TG are activated during acute itch processing

**FIGURE 2:**
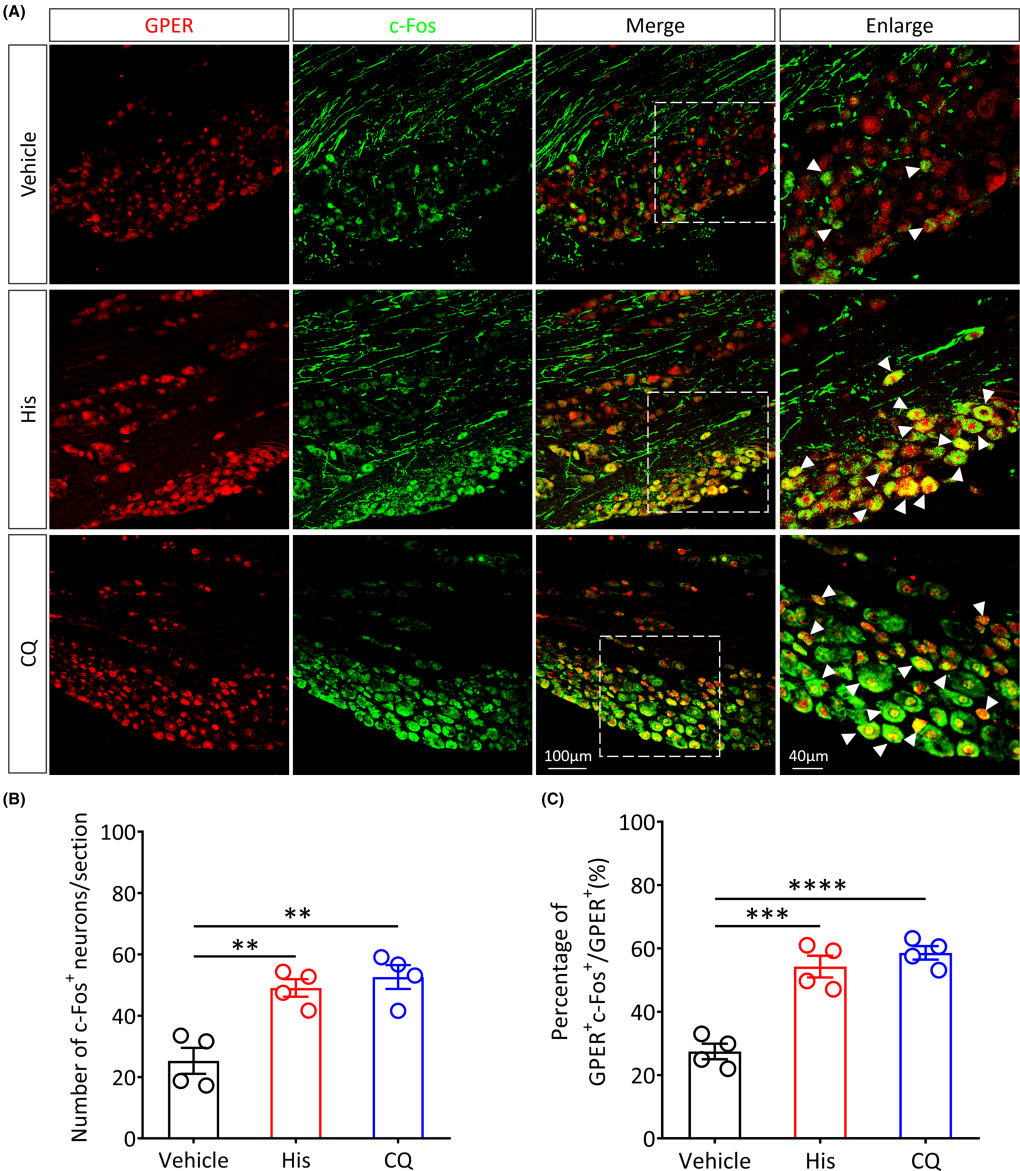
GPER^+^ neurons in the TG of male mice are significantly activated by acute itch stimuli. (A) Representative immunofluorescence images showing the colocalization of GPER (red) and c‐Fos (green) in TG after administration of vehicle (saline), histamine, or chloroquine (scale bar: 100 or 40 μm). (B) Quantitative analysis of the number of c‐Fos^+^ neurons in TG after acute itch stimuli (*n* = 4 per group, **p < 0.01, one‐way ANOVA with Tukey's post hoc test). (C) Quantitative analysis of the percentage of activated GPER^+^ neurons in total GPER^+^ neurons in the TG subjected to acute itch stimuli (*n* = 4 mice per group, ***p < 0.001, ****p < 0.0001, one‐way ANOVA with Tukey's post hoc test).

To test whether TG GPER^+^ neurons are involved in the transmission of acute itch and to observe the changes in TG GPER^+^ neuron activation, we intradermally injected histamine, chloroquine, or vehicle (saline), into the cheek of wild‐type (WT) mice. To detect the colocalization of c‐Fos (a biomarker of neuronal activation) with the GPER, we used immunofluorescence staining (Figures 2A and S1A). Results demonstrated that, in comparison with the control group, acute chemical itch stimulation evoked by histamine and chloroquine markedly increased the number of c‐Fos‐positive (c‐Fos^+^) neurons in the TG of male mice (Vehicle vs. His, *p* < 0.01; Vehicle vs. CQ, *p* < 0.01; Figure 2B) and female mice (Vehicle vs. His, *p* < 0.0001; Vehicle vs. CQ, *p* < 0.0001; Figure S1B). Moreover, acute chemical itch stimulation significantly upregulated the proportion of activated GPER^+^ neurons in the TG of male mice (Vehicle vs. His, *p* < 0.001; Vehicle vs. CQ, *p* < 0.0001; Figure 2C) and female mice (Vehicle vs. His, *p* < 0.0001; Vehicle vs. CQ, *p* < 0.0001; Figure S1C), indicating that the TG GPER^+^ neurons may play a critical role in the processing of acute itch stimuli. There was no significant difference in the number of activated GPER^+^ neurons in the TG between male and female mice after acute stimulation (*p* > 0.05; Figure S2).

### 3.3 GPER ^+^ neurons of the TG are activated under chronic itch conditions

**FIGURE 3:**
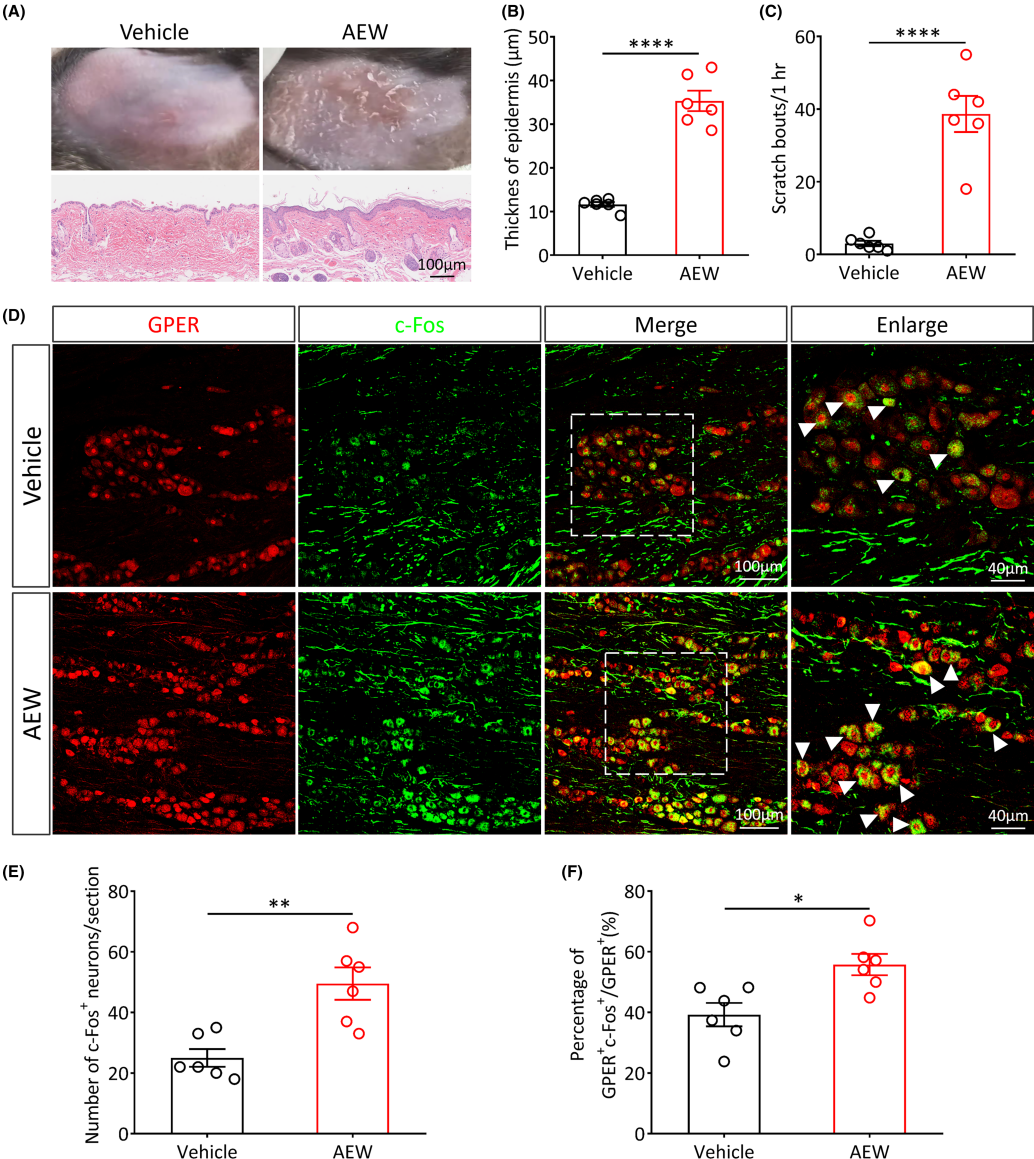
GPER^+^ neurons in the TG of male mice are significantly activated under acetone, ether, and water (AEW)‐induced chronic itch condition. (A) Representative photographs (top) and hematoxylin and eosin (HE) staining images (bottom) of the cheek, before and after AEW treatment in WT mice. Scale bar: 100 μm. (B) The thickness of cheek skin epidermis in vehicle‐ and AEW‐treated WT mice (*n* = 6 per group, ****p < 0.0001, unpaired Student's *t‐*test). (C) The spontaneous scratching behavior was significantly increased in AEW‐treated mice, in comparison with the vehicle group (*n* = 6 per group, ****p < 0.0001, unpaired Student's *t‐*test). (D) Representative immunofluorescence images showing the colocalization of GPER (red) and c‐Fos (green) in TG of vehicle‐ and AEW‐treated mice (scale bar: 100 or 40 μm). (E) Quantitative analysis of the number of c‐Fos^+^ neurons in TG under AEW‐induced chronic itch conditions (*n* = 6 per group, **p < 0.01, unpaired Student's *t‐*test). (F) Quantitative analysis of the percentage of activated GPER^+^ neurons in total GPER^+^ neurons of the TG under AEW‐induced chronic itch conditions (*n* = 6 per group, *p < 0.05, unpaired Student's *t*‐test).

To investigate whether GPER^+^ neurons of the TG are involved in the regulation of chronic itch perception, we established a mouse cheek AEW chronic itch model.^30^ The AEW model induced significant dryness of the skin (Figure 3A) and increased the epidermal thickness (Vehicle vs. AEW, *p* < 0.0001; Figure 3B). In comparison with the vehicle group, the spontaneous scratching behavior was significantly increased (Vehicle vs. AEW, *p* < 0.0001; Figure 3C) and the wiping behavior (related to pain) was not significantly changed (Vehicle vs. AEW, *p* > 0.05; Figure S3) in AEW‐treated mice. Immunofluorescence results showed that, in comparison with the vehicle group, the number of c‐Fos^+^ neurons was markedly increased in the TG of male AEW‐treated mice (Vehicle vs. AEW, *p* < 0.01; Figure 3D,E) and female AEW‐treated mice (Vehicle vs. AEW, *p* < 0.001; Figure S4A,B). Further analysis indicated that the AEW‐induced chronic itch increased the proportion of activated GPER^+^ neurons in the TG of male mice (Vehicle vs. AEW, *p* < 0.05; Figure 3F) and female mice (Vehicle vs. AEW, *p* < 0.01; Figure S4C), indicating that these neurons are involved in the modulation of AEW‐induced chronic itch. There was no significant difference in the number of activated GPER^+^ neurons in the TG between male and female mice under chronic itch conditions (*p* > 0.05, Figure S5).

### 3.4 Chemogenetic activation or inhibition of TG GPER ^+^ neurons differentially modulates itch‐related behaviors

**FIGURE 4:**
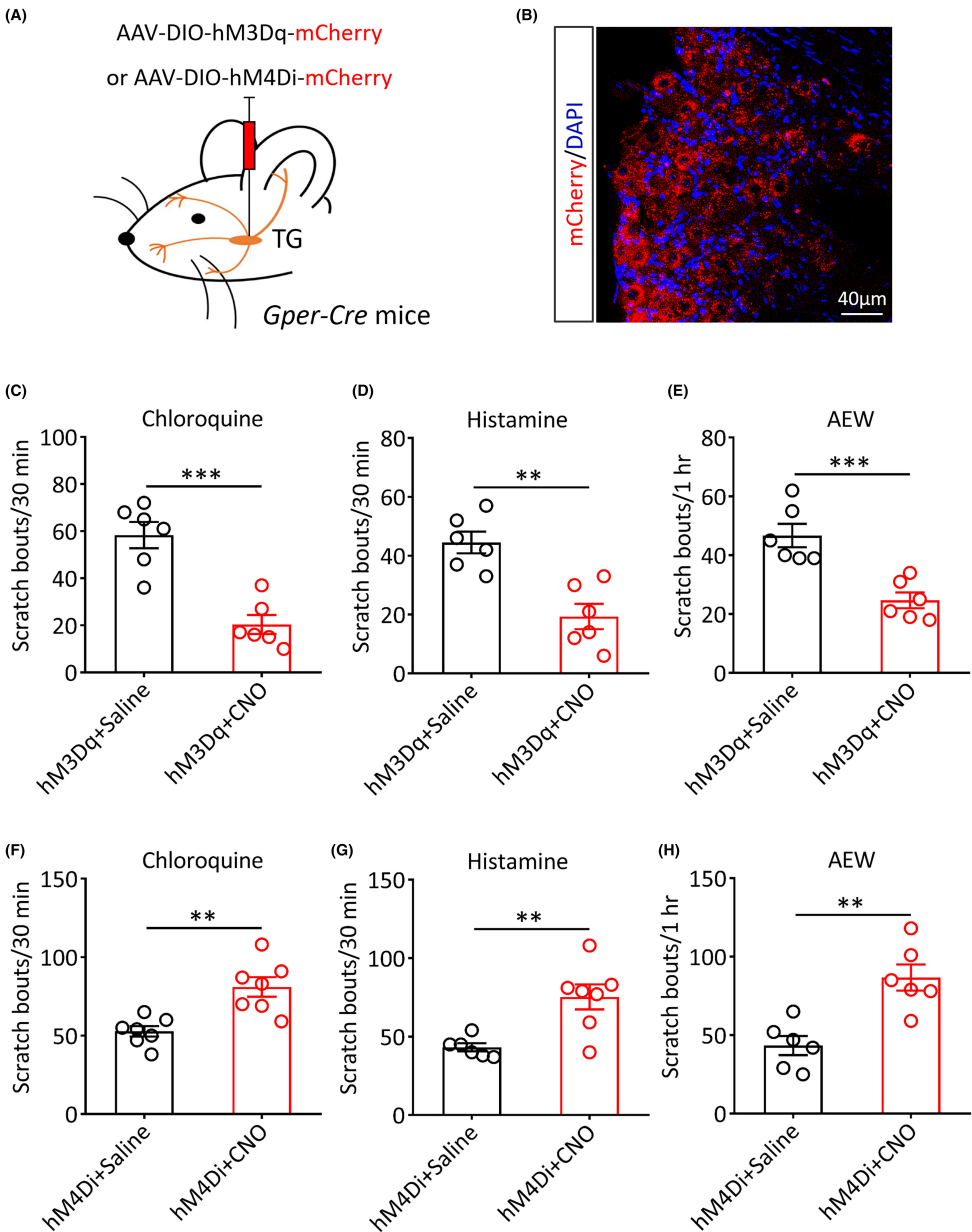
The effects of chemogenetic activation or inhibition of TG GPER^+^ neurons on scratching behavior in *Gper‐Cre* mice. (A) Schematic showing the microinjection of a chemogenetic virus into the TG of *Gper‐Cre* mice. (B) A representative image showing the histological verification of successful viral transduction in TG neurons (red, scale bar: 40 μm). (C, D) Effects of the chemogenetic activation of TG GPER^+^ neurons on the scratching behavior induced by chloroquine (C) and histamine (D) (*n* = 6 per group, **p < 0.01, ***p < 0.001, unpaired Student's *t*‐test). (E) Effects of the chemogenetic activation of TG GPER^+^ neurons on the spontaneous scratching behavior in acetone, ether, and water (AEW) mice (*n* = 6 per group, ***p < 0.001, unpaired Student's *t*‐test). (F, G) Chemogenetic inhibition of TG GPER^+^ neurons increased scratching behavior induced by chloroquine (F) and histamine (G) (*n* = 6 or 7 per group, **p < 0.01, unpaired Student's *t‐*test). (H) The chemogenetic inhibition of TG GPER^+^ neurons increased spontaneous scratching behavior in AEW‐treated mice (*n* = 6 per group, **p < 0.01, unpaired Student's *t*‐test).

Next, to further clarify the exact role of the TG GPER^+^ neurons in itch modulation, we used a chemogenetic approach to specifically activate or suppress these neurons and observe the impact on itch‐related scratching behaviors. The adeno‐associated virus (AAV) containing the excitatory (hM3Dq) or inhibitory (hM4Di) designer receptor fused with mCherry was microinjected into the TG of *Gper‐Cre* mice (Figure 4A). The successful transduction and functional effects of virus in TG neurons were detected by immunofluorescence staining (Figures 4B and S6). Itch‐related behaviors were monitored 30 min after the administration of the hM3Dq or hM4Di agonist clozapine‐N‐oxide (CNO) or saline solution. Behavioral data showed that the scratch bouts evoked by CQ (hM3Dq + saline: 58.33 ± 5.59 vs. hM3Dq + CNO: 20.33 ± 4.03, *p* < 0.001; Figure 4C) and histamine (hM3Dq + saline: 44.50 ± 3.70 vs. hM3Dq + CNO: 19.33 ± 4.33, *p* < 0.01; Figure 4D) were both significantly attenuated following the chemogenetic activation of TG GPER^+^ neurons. Similarly, in comparison with the negative control, the scratching behavior in AEW‐treated mice was significantly decreased after administration of CNO (hM3Dq + saline: 46.67 ± 3.96 vs. hM3Dq + CNO: 24.67 ± 2.69, *p* < 0.001; Figure 4E). We next investigated the effects of the chemogenetic inhibition of TG GPER^+^ neurons on itch‐related behavior. We found that the chemogenetic inhibition of these neurons by CNO administration significantly increased the scratching behavior induced by chloroquine (hM4Di + saline: 52.71 ± 3.34 vs. hM4Di + CNO: 81.00 ± 6.21, *p* < 0.01; Figure 4F) and histamine (hM4Di + saline: 43.17 ± 2.57 vs. hM4Di + CNO: 75.29 ± 8.00, *p* < 0.01; Figure 4G). Similarly, the scratching behavior in AEW mice was significantly increased following the chemogenetic inhibition of TG GPER^+^ neurons (hM4Di + saline: 43.33 ± 6.06 vs. hM4Di + CNO: 86.67 ± 8.35, *p* < 0.01; Figure 4H). Together, the above results suggest that the GPER^+^ neurons in the mouse TG can negatively regulate acute and chronic itch‐induced scratching behavior.

### 3.5 GPER signaling is upregulated in TG neurons under chronic itch conditions

**FIGURE 5:**
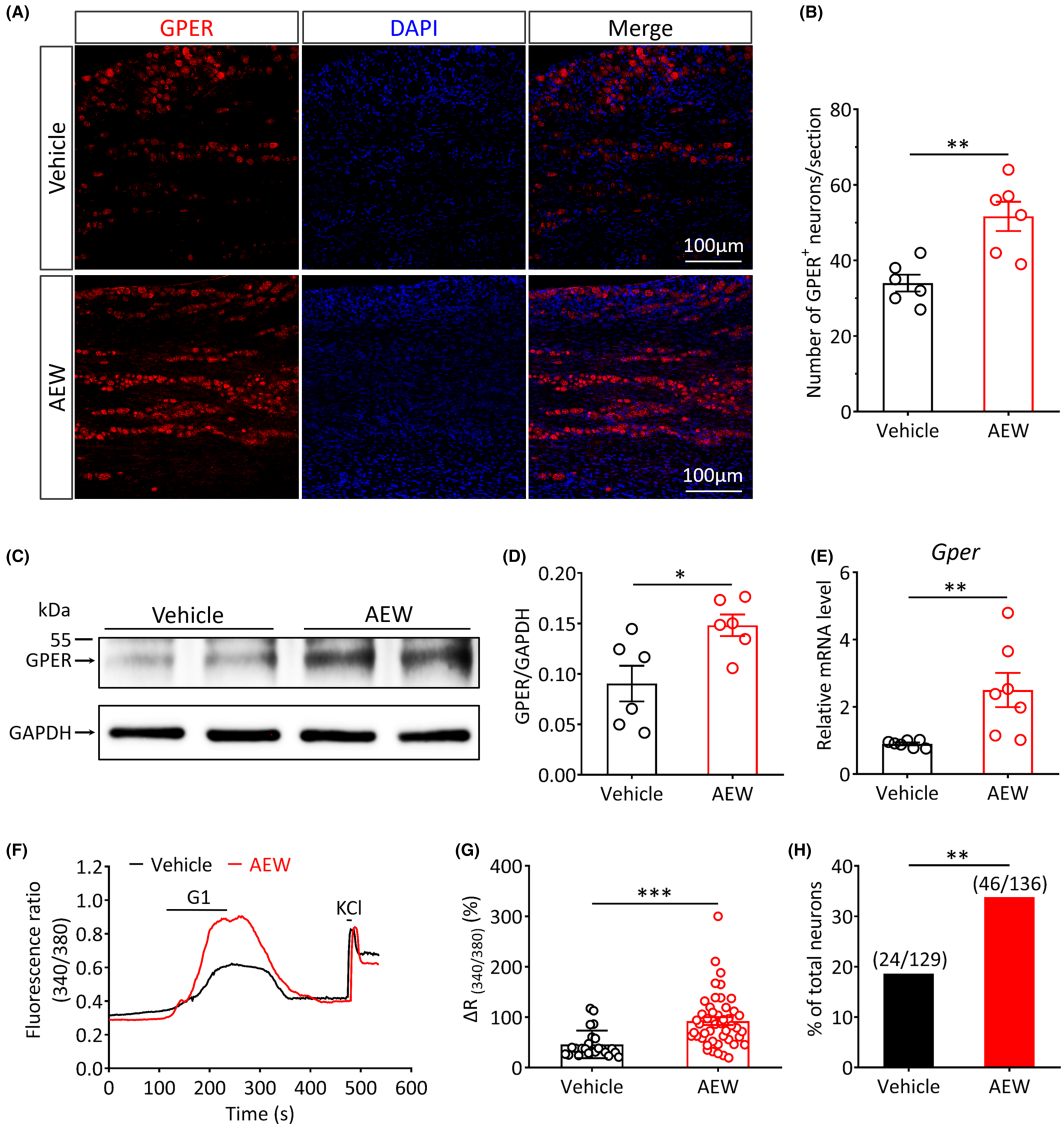
Expression and function of the GPER in TG neurons are both upregulated under AEW‐induced chronic itch conditions. (A) Representative immunofluorescence images showing the colocalization of GPER (red) in the TG of the vehicle‐ and AEW‐treated mice (scale bar: 100 μm). (B) Quantitative analysis of the number of GPER^+^ neurons in the TG of vehicle‐ and AEW‐treated mice (*n* = 6 per group, **p < 0.01, unpaired Student's *t‐*test). (C) Representative western blot bands showing the expression of the GPER protein in the TG of vehicle‐ and AEW‐treated mice (MW: GPER, 52 kDa; GAPDH, 36 kDa). (D) Statistical analysis revealed a significant increase in the protein expression levels of GPER in AEW‐treated mice (*n* = 6 per group, *p < 0.05, unpaired Student's *t‐*test). (E) The relative mRNA expression levels of GPER in the TG of vehicle‐ and AEW‐treated mice (*n* = 7 per group, **p < 0.01, unpaired Student's *t*‐test). (F) Representative traces of Ca^2+^ responses evoked by G1 (10 μM) in TG neurons of the vehicle‐ (black) and AEW‐treated (red) mice. Black bars above the traces show the duration of the chemical treatment. (G) The G1‐induced Ca^2+^ signal amplitude was significantly higher in AEW‐treated mice than in vehicle‐treated mice (***p < 0.001, unpaired Student's *t*‐test). (H) In comparison with the vehicle group, the proportion of G1‐responsive neurons was significantly increased in the AEW‐treated group (**p < 0.01, χ^2^ test).

Next, we hypothesized whether the expression levels and function of the GPER of the TG were altered under chronic itch conditions. To address this hypothesis, we first examined the GPER expression levels in the TG under chronic itch conditions. Immunofluorescence staining revealed that the AEW‐induced chronic itch led to a significant upregulation of the number of GPER^+^ neurons in the TG (Vehicle vs. AEW, *p* < 0.01; Figure 5A,B). Western blot analysis revealed that, in comparison with the control group, the protein expression levels of the GPER in the TG were markedly increased in the AEW‐treated mice (Vehicle vs. AEW, *p* < 0.05; Figure 5C,D). In addition, RT‐qPCR analysis showed that, in comparison with the vehicle group, the mRNA expression levels of *Gper* were significantly increased in the AEW‐treated mice (Vehicle vs. AEW, *p* < 0.01; Figure 5E). We further investigated whether the GPER function in TG neurons was upregulated under chronic itch conditions. As such, we compared calcium responses evoked by G1 (GPER agonist, 10 μM)^45^ in TG neurons, using AEW‐treated and vehicle‐treated mice. The results showed that the amplitude of the G1‐evoked calcium responses in AEW‐treated mice was higher than that of control mice (Vehicle vs. AEW, *p* < 0.001; Figure 5F,G). Further analysis found that the proportion of G1‐responsive neurons was significantly increased in the AEW‐treated mice (Vehicle vs. AEW, *p* < 0.01; Figure 5H). These results indicate that the expression levels and function of GPER in the TG are upregulated under AEW‐induced chronic itch conditions. These observations indicate that GPER signaling may play an essential role in chronic itch regulation.

### 3.6 *Gper* deficiency results in itch hypersensitivity in mice

**FIGURE 6:**
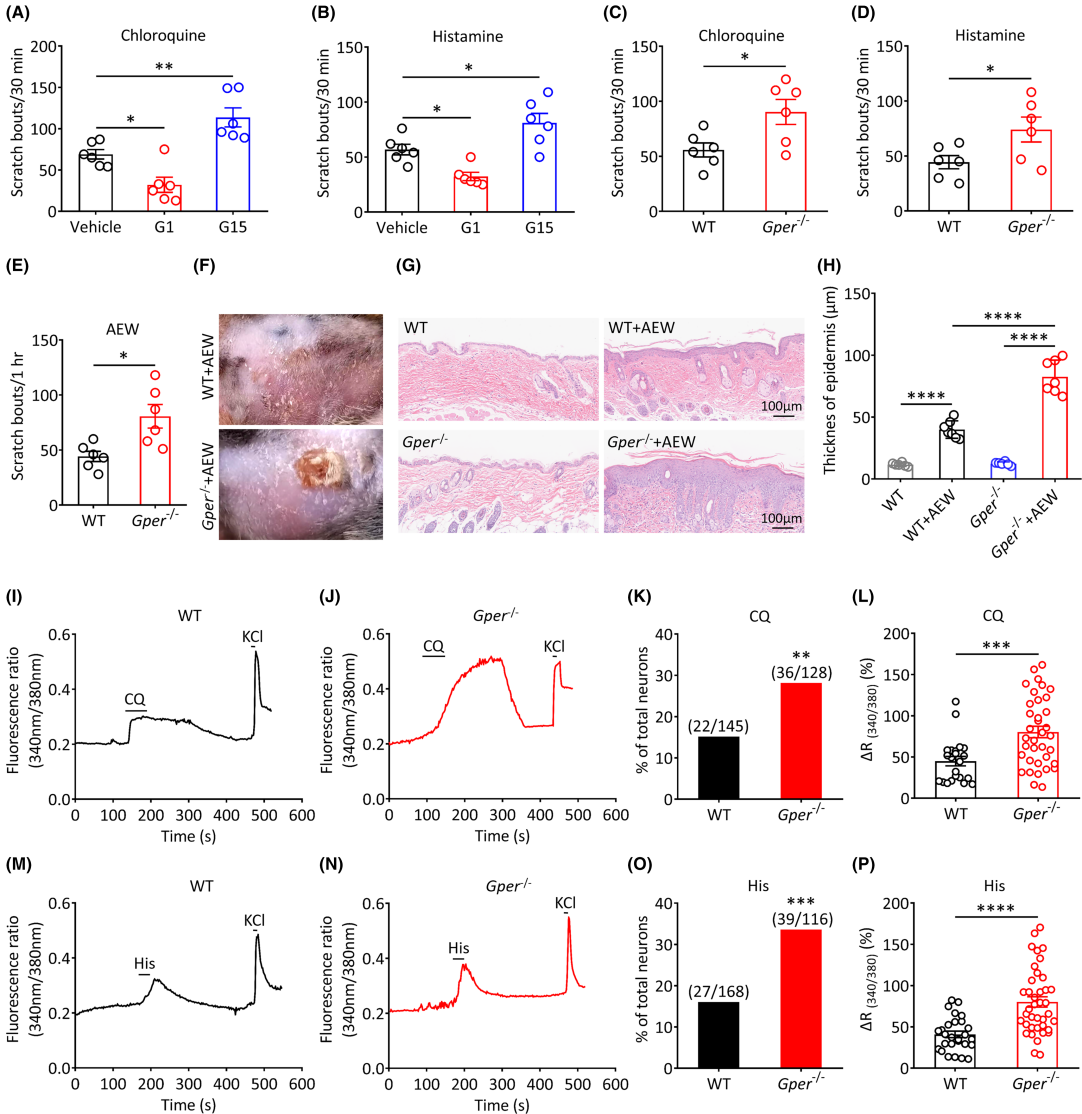
*Gper* knockout in mice results in itch hypersensitivity. (A, B) Administration of G1 significantly decreased the number of scratch bouts induced by chloroquine or histamine. Administration of G15 significantly increased the number of scratch bouts induced by chloroquine or histamine (*n* = 6 per group, *p < 0.05, **p < 0.01, one‐way ANOVA with Tukey's post hoc test). (C–E) Scratching behavior of WT and *Gper* ^−/−^ mice induced by chloroquine (C), histamine (D) and AEW (E) (*n* = 6 per group, *p < 0.05, unpaired Student's *t*‐test). (F) Representative photographs of the cheek after AEW treatment in WT and *Gper* ^−/−^ mice. (G) Representative HE staining images of cheek skin before and after AEW treatment in WT and *Gper* ^−/−^ mice (scale bar: 100 μm). (H) The thickness of the epidermis of the cheek skin in naive and AEW‐treated WT and *Gper* ^−/−^ mice (*n* = 6 or 7 per group, ****p < 0.0001, one‐way ANOVA with Tukey's post hoc test). (I, J) Representative traces of Ca^2+^ responses evoked by 1 mM CQ in TG neurons of WT mice (I) and *Gper* ^−/−^ mice (J). (K) In comparison with WT mice, the proportion of CQ‐responsive neurons was significantly increased in the TG of *Gper* ^−/−^ mice (**p < 0.01, χ^2^ test). (L) In comparison with WT mice, the Ca^2+^ signal amplitude induced by CQ was significantly higher in *Gper* ^−/−^ mice (***p < 0.001, unpaired Student's *t*‐test). (M, N) Representative traces of Ca^2+^ responses evoked by 100 μM His in TG neurons of WT mice (M) and *Gper* ^−/−^ mice (N). (O) In comparison with WT mice, the proportion of His‐responsive neurons was significantly increased in the TG of *Gper* ^−/−^ mice (***p < 0.001, χ^2^ test). (P) In comparison with WT mice, the Ca^2+^ signal amplitude induced by His was significantly higher in *Gper* ^−/−^ mice (****p < 0.0001, unpaired Student's *t*‐test).

To further investigate the role of GPER signaling in acute and chronic itch perception, we examined the effects of the GPER pharmacological activation or inhibition on itch‐related behaviors. After intradermal administration of G1 into the cheek of WT mice, the scratching behavior induced by chloroquine (Vehicle vs. G1, *p* < 0.05; Figure 6A) and histamine (Vehicle vs. G1, *p* < 0.05; Figure 6B) was significantly decreased. In contrast, administration of G15, an antagonist of GPER, significantly increased the scratching behavior induced by chloroquine (Vehicle vs. G15, *p* < 0.01; Figure 6A) and histamine (Vehicle vs. G15, *p* < 0.05; Figure 6B).

To further investigate the role of GPER signaling in acute and chronic itch perception, we used *Gper* knockout (*Gper* ^*−*/−^) mice. In comparison with WT mice, the number of scratch bouts evoked by chloroquine (WT: 55.83 ± 6.38 vs. *Gper* ^−/−^: 90.33 ± 11.36, *p* < 0.05; Figure 6C) and histamine (WT: 44.33 ± 6.00 vs. *Gper* ^−/−^: 74.00 ± 11.32, *p* < 0.05; Figure 6D) was significantly increased in *Gper* ^−/−^ mice. Similarly, the spontaneous scratching behavior was also increased in *Gper* ^−/−^ mice treated with AEW (WT: 44.17 ± 4.64 vs. *Gper* ^−/−^: 80.67 ± 10.71, *p* < 0.05; Figure 6E). After spontaneous scratching behavior was detected, the skin on the scratched area of the cheek was visually compared (Figure 6F), and HE staining was performed on the damaged skin (Figure 6G). The skin lesion of the *Gper* ^−/−^ mice was more severe than that of WT mice. The epidermal thickness of *Gper* ^−/−^ mice was thicker than that of WT mice (WT + AEW vs. *Gper* ^−/−^ + AEW, *p* < 0.0001; Figure 6H), likely because of more intense scratching activity.

Next, using *Gper* ^−/−^ mice, we further identified alterations in the calcium response of TG neurons to chloroquine and histamine. Calcium imaging results showed that, in comparison with WT mice, the proportion of chloroquine‐responsive TG neurons was markedly increased in *Gper* ^−/−^ mice (WT vs. *Gper* ^−/−^, *p* < 0.01; Figure 6I–K). Furthermore, the amplitude of the chloroquine‐evoked calcium response was also significantly increased (WT vs. *Gper* ^−/−^, *p* < 0.01; Figure 6L). Similarly, in comparison with WT mice, more TG neurons were responsive to histamine in *Gper* ^−/−^ mice (WT vs. *Gper* ^−/−^, *p* < 0.001; Figure 6M–O). The amplitude of the histamine‐induced calcium response was also significantly increased (WT vs. *Gper* ^−/−^, *p* < 0.0001; Figure 6P). In summary, these results reveal that *Gper* deficiency results in hypersensitivity to itch in mouse.

### 3.7 *Gper* deficiency enhances the function of the TRPA1 and TRPV1 in TG neurons

**FIGURE 7:**
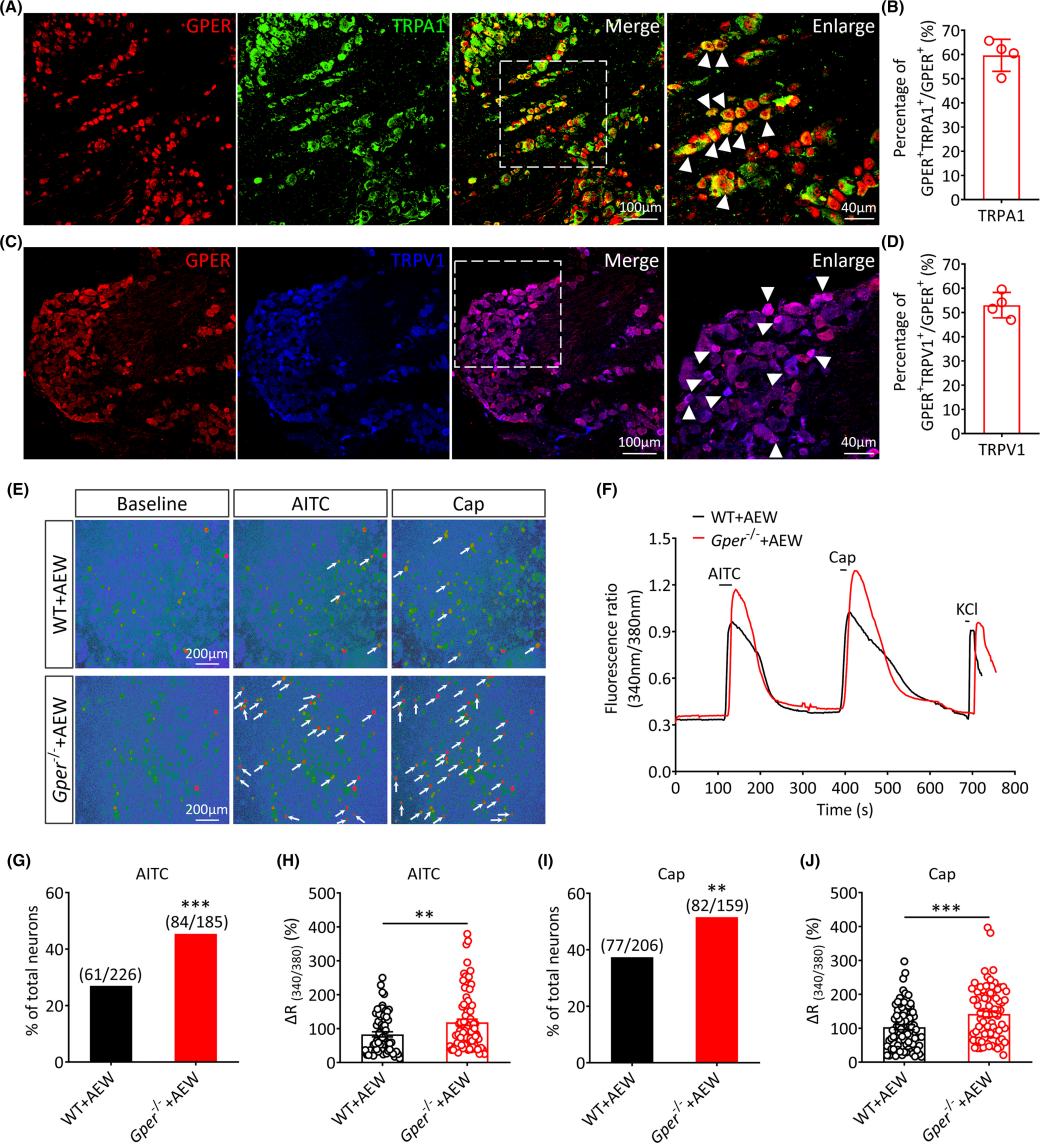
*Gper* deficiency enhances the function of TRPA1 and TRPV1 in TG neurons. (A) Representative immunofluorescence images showing the colocation of GPER with TRPA1. (B) Quantitative analysis of the percentage of GPER^+^/TRPA1^+^ neurons in total GPER^+^ neurons of the TG (*n* = 4 mice per group, scale bar: 100 or 40 μm). (C) Representative immunofluorescence images showing colocation of GPER and TRPV1. (D) Quantitative analysis of the percentage of GPER^+^/TRPV1^+^ neurons in total GPER^+^ neurons of the TG (*n* = 4 mice per group, scale bar: 100 or 40 μm). (E) Representative fluorescence images of mouse TG neurons loaded with Fura‐2 at the baseline and after application of allyl isothiocyanate (AITC, 100 μM) or capsaicin (Cap, 1 μM). Arrows indicate TG neurons responsive to the agent (scale bar: 200 μm). (F) Representative traces of Ca^2+^ responses evoked by AITC or Cap in TG neurons of WT (black) and *Gper* ^−/−^ (red) mice. Black bars above the traces show the duration of the chemical treatment. (G–J) In comparison with WT mice, the percentage of AITC‐responsive (G) or Cap‐responsive neurons (I) was significantly increased in *Gper* ^−/−^ mice (**p < 0.01, ***p < 0.001, χ^2^ test). In comparison with WT mice, the Ca^2+^ signal amplitude induced by AITC (H) or Cap (J) was significantly higher in *Gper* ^−/−^ mice (**p < 0.01, ***p < 0.001, unpaired Student's *t‐*test).

According to previous studies, the peripheral TRPA1 and TRPV1 are the two main ion channels required for itch perception, mediating the nonhistaminergic and histaminergic itch perception, respectively.^46^ TRPA1 and TRPV1 are also considered to play a key role in the AEW‐induced chronic itch behavior.^30^, ^47^ Thus, we hypothesized whether the GPER of the TG modulates the itch sensation by regulating the functions of TRPA1 and TRPV1. To address this hypothesis, we initially characterized the colocalization extent of GPER with TRPA1 or TRPV1. Immunofluorescence results showed that approximately 59.64 ± 3.32% of GPER^+^ neurons expressed TRPA1 (Figure 7A,B), while 53.03 ± 2.62% of GPER^+^ neurons expressed TRPV1 (Figure 7C,D). These results offer a histological basis for a possible contribution of the TRP channels in the regulation of itch sensation by the GPER in TG neurons.

Next, we further clarified whether *Gper* deficiency affects the function of the TRPA1 and TRPV1 in TG neurons under the AEW‐induced chronic itch condition. We established AEW‐induced chronic itch models in WT and *Gper* ^−/−^ mice, and then performed calcium imaging in isolated TG neurons (Figure 7E). Calcium imaging results showed that the proportion of AITC (TRPA1 agonist; 100 μM)‐responsive neurons in *Gper* ^−/−^ mice was higher than in WT mice (WT + AEW vs. *Gper* ^−/−^ + AEW, *p* < 0.001; Figure 7F,G). The amplitude of the calcium response to AITC was also increased (WT + AEW vs. *Gper* ^−/−^ + AEW, *p* < 0.01; Figure 7H). A similar phenomenon was observed when TG neurons were treated with the TRPV1 agonist capsaicin (Cap, 1 μM). In comparison with WT mice, a significantly higher proportion of Cap‐responsive TG neurons was detected in *Gper* ^−/−^ mice (WT + AEW vs. *Gper* ^−/−^ + AEW, *p* < 0.01; Figure 7F,I). Additionally, the amplitude of the calcium response to Cap was also greater in *Gper* ^−/−^ mice than in WT mice (WT + AEW vs. *Gper* ^−/−^ + AEW, *p* < 0.001; Figure 7J). These results indicate that *Gper* deficiency can upregulate the function of TRPA1 and TRPV1 in TG neurons, which may be the underlying mechanism by which the GPER regulates the itch sensation.

## 4 DISCUSSION

The molecular mechanisms of itch perception processing in the TG are remarkably complex. Our recent findings suggest that the GPER, originally known for its role in the modulation of pain perception, may also be important in the regulation of the itch sensation.^26^, ^48^, ^49^ The current study has provided evidence that the GPER‐expressing neurons of the TG are antipruritic neurons. These neurons are mobilized under acute and chronic itch conditions, possibly acting through the GPER‐dependent inhibition of TRPA1 and TRPV1 signaling. These findings provide new insights into the molecular mechanisms of peripheral itch sensory signal processing and identify the GPER as a potential target for chronic itch treatment.

In previous studies, GPER expression has been detected in many organ areas such as the hypothalamus, the midbrain^23^, ^50^ and the spinal cord,^51^ as well as in peripheral tissues.^52^ Growing evidence suggests that the GPER plays multiple roles in the central and peripheral nervous systems of rodents.^45^, ^48^, ^49^, ^53^ However, no studies on the regulation of itch signaling by the GPER in the peripheral nervous system are available. Neurons of the TG can be peptidergic or nonpeptidergic.^54^, ^55^ We tested the cellular localization of GPER‐expressing neurons in the TG by double immunofluorescence staining with IB4, CGRP and NF200. Results revealed that the GPER was mainly expressed in small‐diameter peptidergic or non‐peptidergic neurons of the TG. We also found that the mouse cheek is innervated by TG GPER^+^ neurons. Thus, the primary objective of this study was to determine whether the GPER of the TG would be related to the transmission of the facial itch sensation.

After administration of the pruritogenic agents' histamine or chloroquine into the mouse cheek, most of the TG GPER^+^ neurons were activated, suggesting that these neurons may be involved in the modulation of acute itch perception. Moreover, in comparison with the negative control, we found higher levels of activated GPER^+^ neurons in the TG in the AEW‐induced chronic itch mouse model. This finding suggests that these neurons are also engaged in regulating chronic itch perception. In order to identify the exact role of the GPER^+^ neurons of the TG in itch perception, we used chemogenetic manipulations to specifically activate GPER^+^ neurons in TG. We found that both acute and chronic itch behavior were significantly suppressed, while the specific inhibition of GPER^+^ neurons remarkably increased scratching behavior. Thus, the GPER^+^ neurons of the TG may exert inhibitory effects on the transmission of facial itch signals. Our current findings are consistent with a previous study from our team, which suggests that GPER^+^ neurons exert important itch‐suppressing effects in the RVM.^26^

Furthermore, we found that both the expression levels and function of the GPER in the TG were upregulated under chronic itch conditions. Consistent with the inhibition effects on GPER^+^ neurons, *Gper* ^−/−^ mice showed significantly increased acute and chronic itch behaviors. Importantly, the number of TG neurons responsive to chloroquine or histamine was significantly increased and the amplitude of calcium response was also enhanced in *Gper* ^−/−^ mice. Thus, we speculate that the GPER signaling in TG neurons may have a negative regulatory function, in both histaminergic and non‐histaminergic itch perception.

There are two major pathways of itch perception: histaminergic and nonhistaminergic.^56^ In general, the activation of histamine receptors is related to the excitation and sensitization of TRPV1.^57^ On the contrary, chloroquine‐evoked nonhistaminergic itch perception is mediated by TRPA1 activation.^21^ In addition, TRP channels are associated with the activation of G protein‐coupled receptors (GPCRs) and the threshold for TRP channel activation is regulated by pruritogen‐activated GPCR signaling.^14^, ^17^ Most pruritogens could activate GPCRs.^58^ We found that the GPER regulated both histaminergic and nonhistaminergic itch perception. Most of the GPER^+^ neurons have shown expression of TRPA1 and TRPV1 in the TG. Therefore, we inferred that, as a GPCR member, the inhibitory effect of the GPER on the pruritogen‐induced facial itch sensation might be achieved by modulating TRPA1 and TRPV1 functions. Consistent with this speculation, calcium imaging results revealed that the percentage of TG neurons responsive to AITC (TRPA1^+^) and capsaicin (TRPV1^+^) was markedly increased in *Gper*‐deficient mice. Moreover, the peak of calcium response was also significantly higher than in control mice. These data imply that the GPER in TG neurons may regulate facial itch signaling by tuning the function of TRPV1 and TRPA1.

In this study, the inhibition of GPER^+^ neurons in the TG enhanced itch signaling, and most GPER^+^ neurons expressed both TRPA1 and TRPV1. Moreover, previous studies revealed that the GPER can promote peripheral nociceptive signaling.^23^, ^44^, ^53^ G protein‐coupled estrogen receptor in TG neurons can mediate trigeminal sensitization to increase facial nociceptive sensation.^44^ Estrogen can trigger migraine through GPER signaling, by regulating the release of vasoactive substances in the TG.^23^ Neurons exerting inhibitory effects on cell signaling processes related to itch perception have been recently discovered.^18^, ^59^ A peripheral neuron subpopulation expressing both TRPA1 and TRPV1 has been shown to have pro‐pain and anti‐itch effects on trigeminal sensory signaling.^60^ Signaling inhibition in these neurons reduces the excitability of the associated inhibitory neurons, further inducing an increase in itch behavior. Therefore, whether GPER^+^ neurons in the TG are a subset of such neurons and the exact molecular mechanisms of how GPER signaling regulates TRP channels under itch conditions are the interesting topics that deserve further investigation.

This study offers a new perspective on the role of GPER in itch perception. Several studies have indicated that the essential role of estrogen in itch behaviors.^61^, ^62^ Therefore, considering the potential effects of estrogenic changes during the estrous cycle on itch behaviors, we used male mice for behavioral tests. Previous studies have shown that GPER expression in the central and peripheral nervous system is similar in male and female rodent.^51^, ^52^ In this study, we found that the number of activated GPER^+^ neurons in the TG of male and female mice was similar under acute and chronic itch conditions. Our previous investigations have shown no gender differences in the modulation of itch by GPER signaling in the RVM.^26^

## 5 CONCLUSIONS

In summary, this study demonstrated the involvement of the GPER of TG in the regulation of acute and chronic itch signaling resulting from stimuli on the mouse cheek. Both the chemogenetic inhibition of GPER^+^ neurons and the knockout of *Gper* enhanced facial itch perception in mouse. The underlying molecular mechanisms may include the upregulation of TRPV1 and TRPA1 functions, which enhance both the acute and chronic itch signals in TG neurons. This study provides new insights into the molecular basis of peripheral itch signal processing and offers a new target for future clinical antipruritic therapy.

## AUTHOR CONTRIBUTIONS

PG, LQY, and XQX were involved in conception and design; JL, PG, and SYZ were involved in acquisition of data; JL, PG, SYZ, XQL, and JHC were involved in data analysis; JL, PG, LQY, XQX. YFJ, SZ, and WFY were involved in manuscript draft and revision, and provided critical suggestions. All authors have read and approved the final manuscript.

## CONFLICT OF INTEREST STATEMENT

The authors declare that they have no competing interests.

## Supporting information


Data S1.

Figures S1‐S6.

## Data Availability

The data that support the findings of this study are available from the corresponding author upon reasonable request.
