# Supplementary figures and images for: The G protein‐coupled estrogen receptor of the trigeminal ganglion regulates acute and chronic itch in mice
Source: CNS Neurosci Ther. 2023 Jul 14;30(2):e14367. doi: 10.1111/cns.14367 (PMC10848076; doi:10.1111/cns.14367)

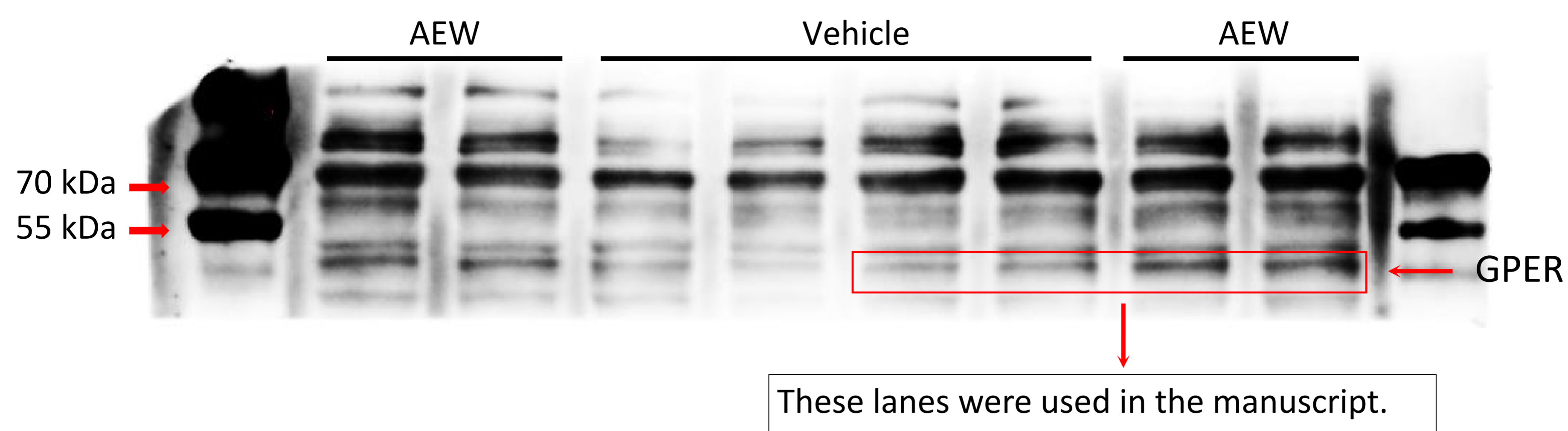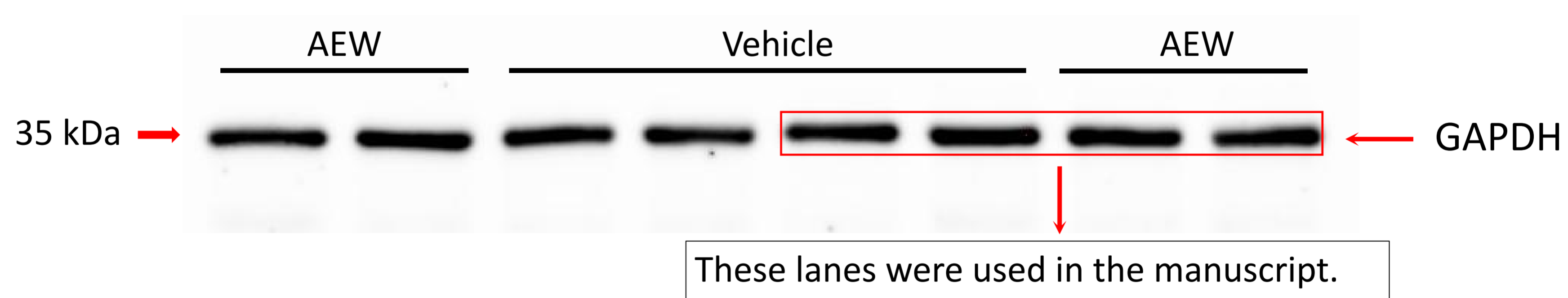

Full unedited blot for Figure 5C

Supplement: Supplementary file 1 — Data S1. [file CNS-30-e14367-s002.pdf]
