# Supplementary material for: The G protein‐coupled estrogen receptor of the trigeminal ganglion regulates acute and chronic itch in mice
Source: CNS Neurosci Ther. 2023 Jul 14;30(2):e14367. doi: 10.1111/cns.14367 (PMC10848076; doi:10.1111/cns.14367)
Supplement: Supplementary file 2 — Figures S1‐S6. [file CNS-30-e14367-s001.docx]

**Supplemental Material**

**The G protein-coupled estrogen receptor of the** **trigeminal ganglion regulates acute and chronic itch in mice**

**Jun Li ^1, 2, 3†^, Po Gao ^2, 3†, *^, Siyu Zhang ^2,^ ^3, 4^, Xiaoqi Lin ^2, 3^, Junhui Chen ^2, 3^, Song Zhang ^2, 3^, Yingfu Jiao ^2, 3^, Weifeng Yu ^2, 3^, Xiaoqiong Xia ^1*^, Liqun Yang ^2, 3*^.**

^1^Department of Anesthesiology, Chaohu Hospital Affiliated to Anhui Medical University, Chaohu, Anhui, 238000, China

^2^Department of Anesthesiology, Renji Hospital, Shanghai Jiao Tong University School of Medicine, Shanghai 200127, China

^3^Key Laboratory of Anesthesiology (Shanghai Jiao Tong University), Ministry of Education, China

^4^Department of Anesthesiology, The Second Affiliated Hospital of Jiaxing University, Jiaxing, 314000, Zhejiang, China

† These authors have contributed equally to this work and share first authorship.

*** Correspondence:**

Po Gao and Liqun Yang, Department of Anesthesiology, Renji Hospital, Shanghai Jiao Tong University School of Medicine, Shanghai, China.

Email: [gaopo0908@163.com](mailto:gaopo0908@163.com) and [lqyang72721@126.com](mailto:lqyang72721@126.com)

Xiaoqiong Xia, Department of Anesthesiology, Chaohu Hospital Affiliated to Anhui Medical University, Chaohu, Anhui, 238000, China.

Email: [xxq2366833@sina.com](mailto:xxq2366833@sina.com)

**Supplemental Figures**


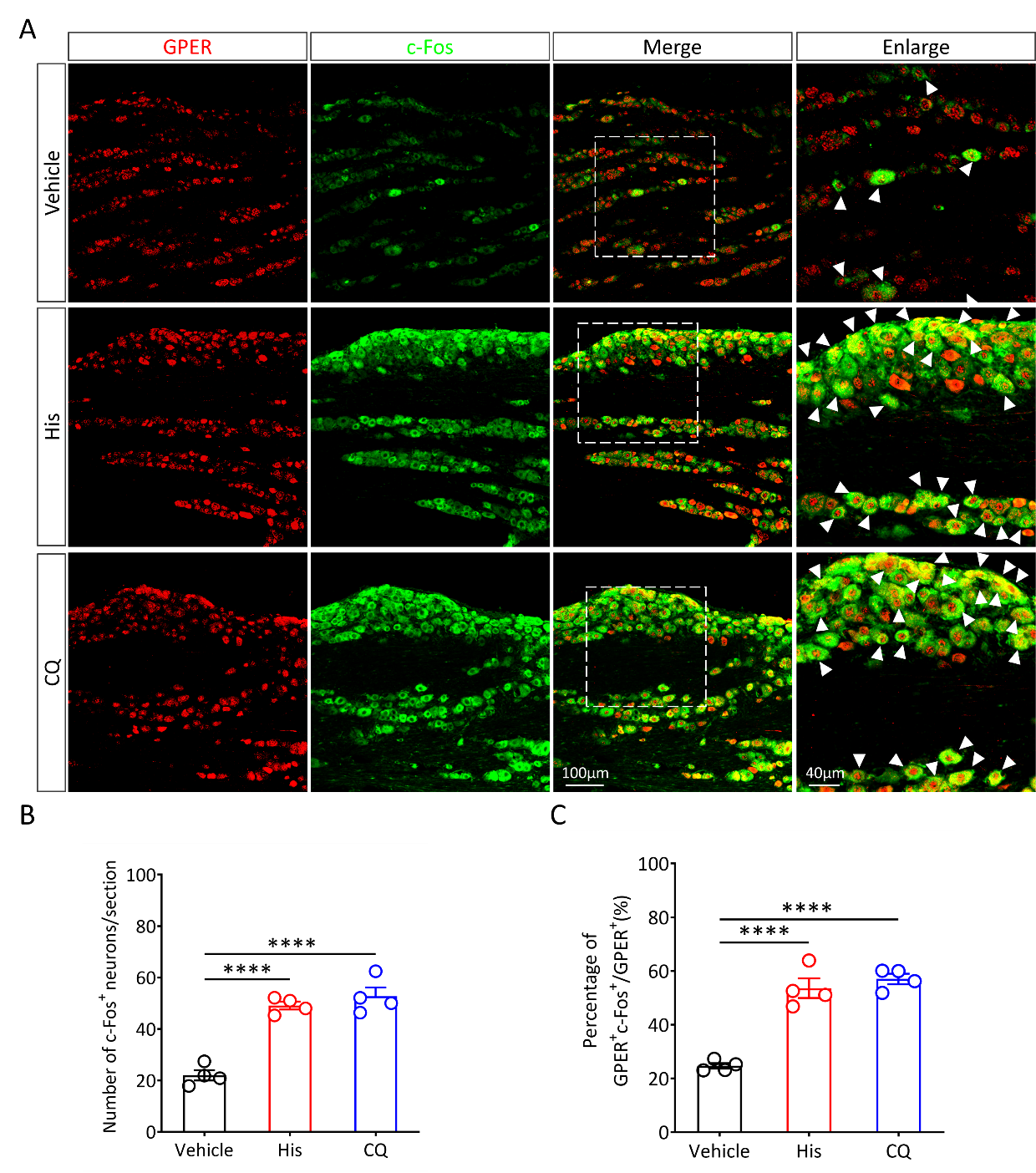


**Supplemental Figure 1.** GPER^+^ neurons in the TG of female mice are significantly activated by acute itch stimuli. **(A)** Representative immunofluorescence images showing the colocalization of GPER (red) and c-Fos (green) in TG after administration of vehicle (saline), histamine, or chloroquine (scale bar: 100 μm or 40 μm). **(B)** Quantitative analysis of the number of c-Fos^+^ neurons in TG after acute itch stimuli (n = 4 per group, *****p* < 0.0001, one-way ANOVA with Tukey’s *post hoc* test). **(C)** Quantitative analysis of the percentage of activated GPER^+^ neurons in total GPER^+^ neurons in the TG subjected to acute itch stimuli (n = 4 mice per group, *****p* < 0.0001, one-way ANOVA with Tukey’s *post hoc* test)

**
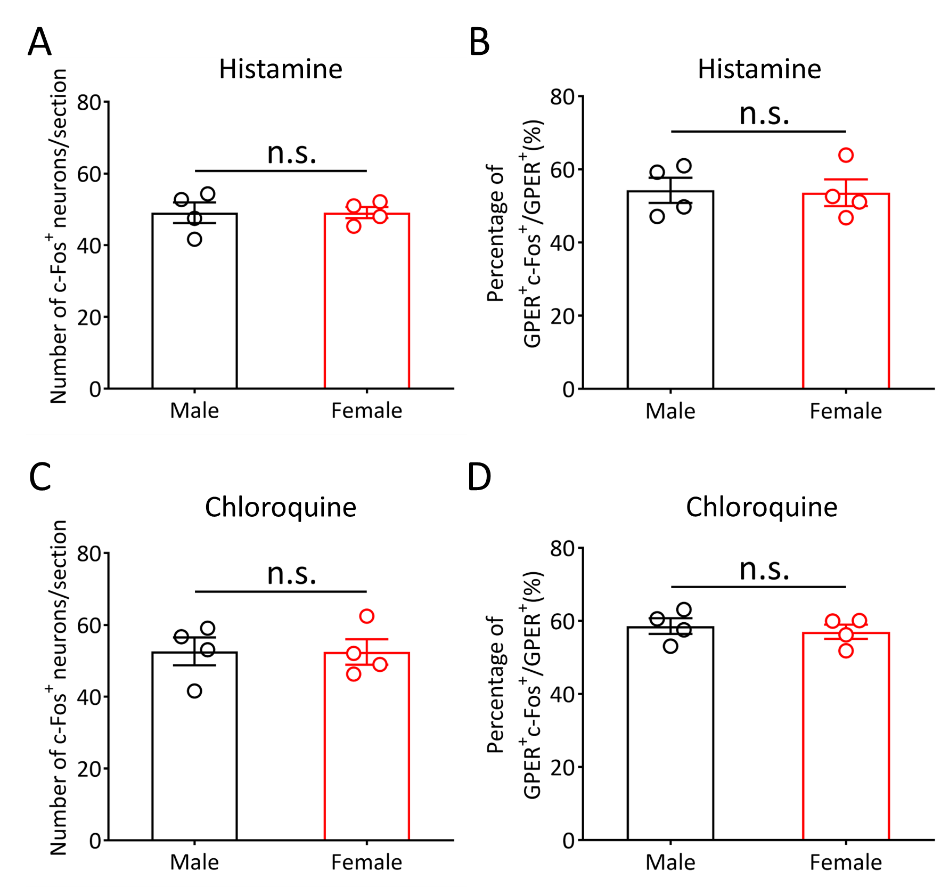
**

**Supplemental Figure 2.** There is no significant difference in the number of activated GPER^+^ neurons in the TG between male and female mice during acute itch processing. **(A, B)** Quantitative analysis of the number of c-Fos^+^ neurons and the percentage of activated GPER^+^ neurons in total GPER^+^ in the TG of male and female mice after histamine stimuli (n = 4 per group, n.s.: no statistical difference, unpaired Student’s *t*-test). **(C, D)** Quantitative analysis of the number of c-Fos^+^ neurons and the percentage of activated GPER^+^ neurons in total GPER^+^ in the TG of male and female mice after chloroquine stimuli (n = 4 per group, n.s.: no statistical difference, unpaired Student’s *t*-test).

**
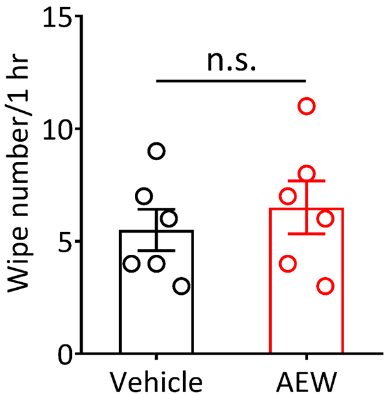
**

**Supplemental Figure 3.** The wiping behavior (related to pain) was not significantly changed in male mice treated with AEW compared to the vehicle group (n = 6 per group, n.s.: no statistical difference, unpaired Student’s *t*-test).

**
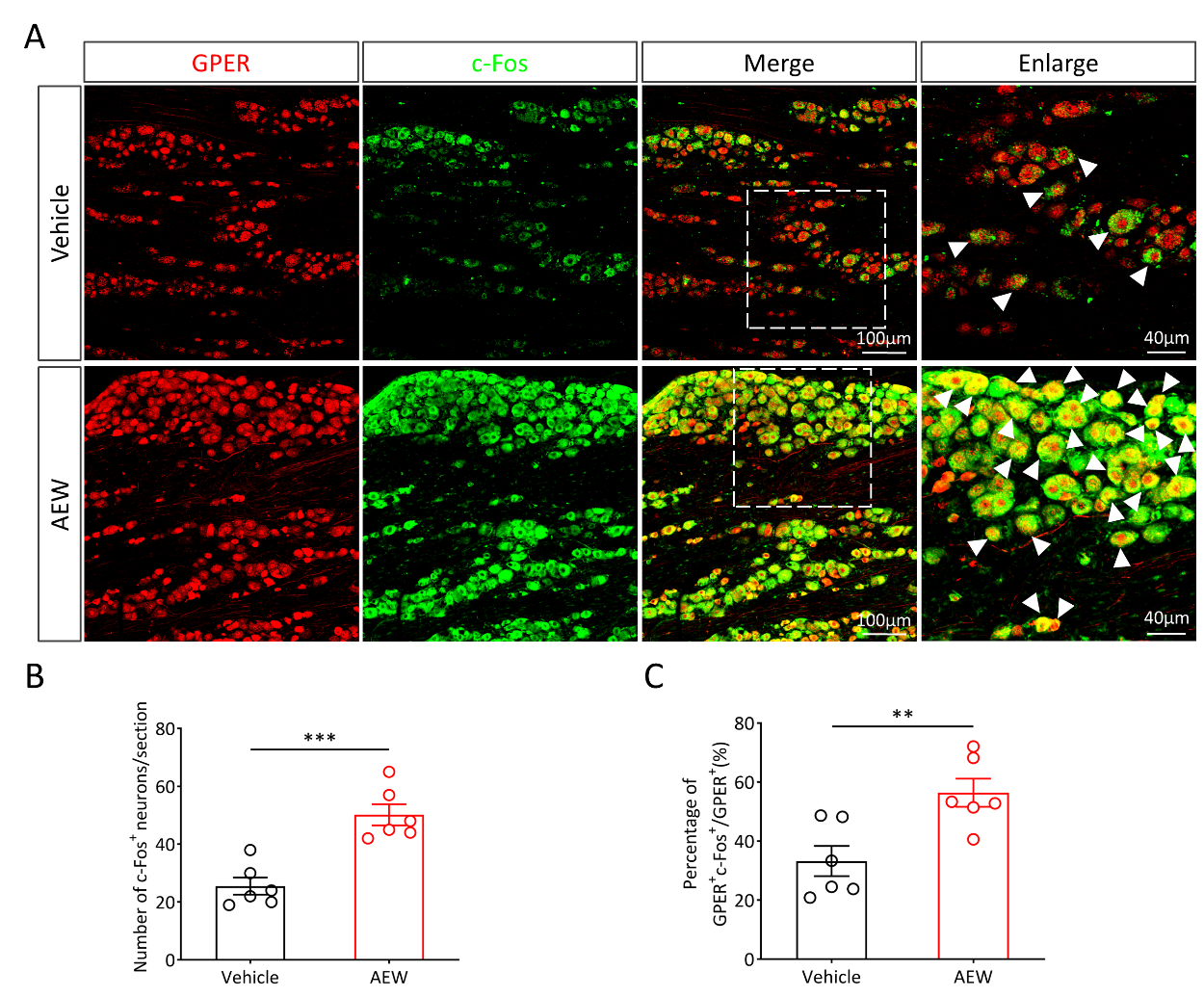
**

**Supplemental Figure 4.** GPER^+^ neurons in the TG of female mice are significantly activated under AEW-induced chronic itch condition. **(A)** Representative immunofluorescence images showing the colocalization of GPER (red) and c-Fos (green) in TG of vehicle- and AEW-treated mice (scale bar: 100 μm or 40 μm). **(B)** Quantitative analysis of the number of c-Fos^+^ neurons in TG under AEW-induced chronic itch conditions (n = 6 per group, ****p* < 0.001, unpaired Student’s *t*-test). **(C)** Quantitative analysis of the percentage of activated GPER^+^ neurons in total GPER^+^ neurons of the TG under AEW-induced chronic itch conditions (n = 6 per group, ***p* < 0.01 unpaired Student’s *t*-test).


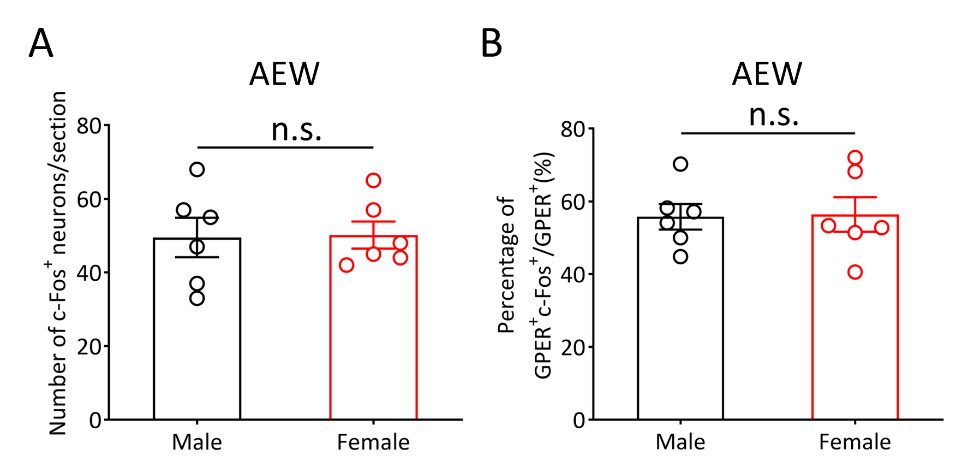


**Supplemental Figure 5.** There is no significant difference in the number of activated GPER^+^ neurons in the TG between male and female mice under chronic itch conditions. **(A)** Quantitative analysis of the number of c-Fos^+^ neurons in TG of male and female mice under AEW-induced chronic itch conditions (n = 6 per group, n.s.: no statistical difference, unpaired Student’s *t*-test). **(B)** Quantitative analysis of the percentage of activated GPER^+^ neurons in total GPER^+^ neurons in TG of male and female mice under AEW-induced chronic itch conditions (n = 6 per group, n.s.: no statistical difference, unpaired Student’s *t*-test).

**
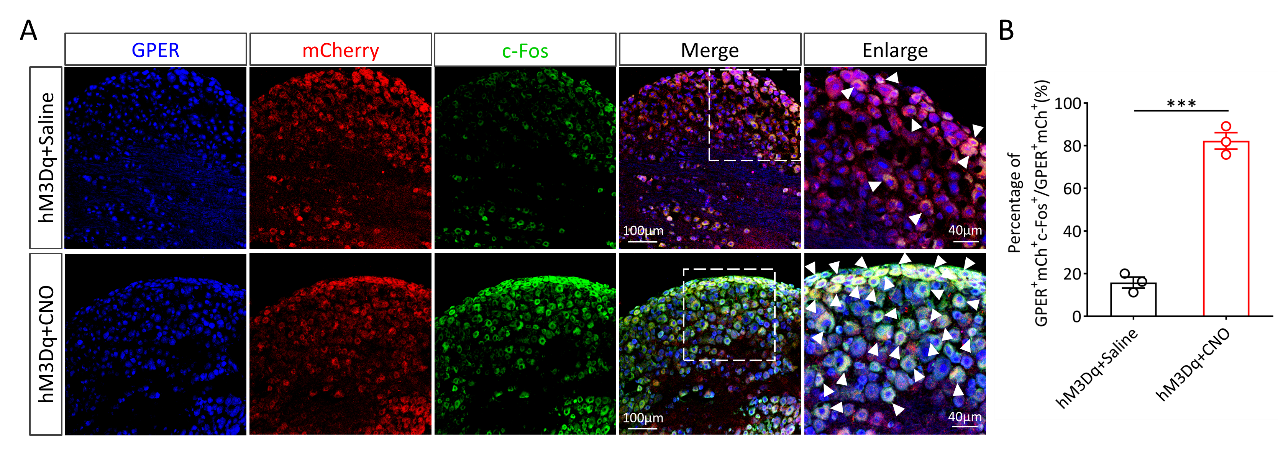
**

**Supplemental Figure 6.** The GPER neurons in the TG were successfully activated by chemogenetic regulation. **(A)** Representative immunofluorescence images showing the colocalization of GPER (blue), mCherry (red) and c-Fos (green) in TG after administration of CNO or saline (scale bar: 100 μm or 40 μm). (B) Quantitative analysis of the percentage of GPER^+^/mCh^+^/c-Fos^+^ neurons in total GPER^+^/mCh^+^ neurons after the administration of CNO or saline (n = 3 per group, ****p* < 0.001, unpaired Student’s *t*-test).
